# Overview of Important Micronutrients Supplementation in Preterm Infants after Discharge: A Call for Consensus

**DOI:** 10.3390/life11040331

**Published:** 2021-04-10

**Authors:** Laura Ilardi, Alice Proto, Federica Ceroni, Daniela Morniroli, Stefano Martinelli, Fabio Mosca, Maria Lorella Giannì

**Affiliations:** 1 Azienda Socio Sanitaria Territoriale Grande Ospedale Metropolitano Niguarda, 20162 Milan, Italy; laura.ilardi@ospedaleniguarda.it (L.I.); alice.proto@ospedaleniguarda.it (A.P.); stefano.martinelli@ospedaleniguarda.it (S.M.); 2 Department of Clinical Sciences and Community Health, University of Milan, 20122 Milan, Italy; federica.ceroni@unimi.it (F.C.); fabio.mosca@unimi.it (F.M.); 3 Fondazione IRCCS Cà Granda Ospedale Maggiore Policlinico, NICU, 20122 Milan, Italy; daniela.morniroli@gmail.com

**Keywords:** complementary feeding, preterm infants, micronutrients

## Abstract

Preterm infants have a lower level of nutrient body stores and immature body systems, resulting in a higher risk of malnutrition. Imbalanced complementary feeding could lead to further risk of nutritional deficits and excesses. However, evidence on their nutritional requirements following hospital discharge is limited. When planning complementary feeding, appropriate micronutrient intake should be considered for their critical role in supporting various body functions. This narrative review summarizes the need for iron, zinc, vitamin D, calcium, phosphate and long-chain polyunsaturated fatty acids (LCPUFAs) supplementation in preterm infants during complementary feeding. Regarding iron and vitamin D, the scientific community is reaching an agreement on supplementation in some categories of prematures. On the contrary, there is still not enough evidence to detail possible recommendations for LCPUFAs, zinc, calcium and phosphorus supplementation. However, these micronutrients are paramount for preterms’ health: LCPUFAs can promote retinal and brain development while calcium and phosphorus supplementation is essential to prevent preterms’ metabolic bone disease (MBD). Waiting for a consensus on these micronutrients, it is clear how the knowledge of the heterogeneity of the prematures population can help adjust the nutritional planning regarding the growth rate, comorbidities and comprehensive clinical history of the preterm infant.

## 1. Introduction

Fetal development and infancy represent a critical time window for shaping future health due to various organs’ extraordinary plasticity and their sensitivity to nutrition and other environmental triggers during this phase [1]. Meeting infants’ nutritional requirements contributes to their optimal growth and development, whereas nutritional deficits, even arising for short periods, may lead to negative long-term health consequences [2]. High nutritional requirements characterize preterm infants (born before 37 weeks of gestational age), both in terms of macronutrients and micronutrients, due to the lower level of nutrients in the body stores at birth, the immaturity of the body systems, the need of rapid postnatal growth and the occurrence of acute illnesses. Adequate and timely preterm infant nutritional support has been advocated to avoid malnutrition and limit postnatal growth retardation, thus preventing the need for rapid catch-up growth, which is associated with late adverse metabolic outcomes [3,4]. Increasing evidence indicates that appropriate nutritional management dramatically reduces the risk of comorbidity development, such as sepsis, metabolic bone disease (MBD) and severe retinopathy of prematurity and the optimization of neurodevelopment outcomes [5,6].

There is a consensus on the preterm infants’ nutritional requirements during the hospital stay, with early enteral and parenteral support being the cornerstone of their nutritional management [7,8]. Specifically, breast milk is recommended as the first option for enteral feeding due to its several health benefits [9,10]. On the contrary, data available on preterm infants’ optimal nutritional management following hospital discharge are limited [11]. An individualized approach, based on the entity and velocity of catch-up growth, the nutritional requirements and the neurodevelopmental and metabolic issues, has been recommended [7]. The complementary feeding period has received limited attention within this context, although it exposes preterm infants at further risk of nutritional deficits and excesses, mainly if they have developed postnatal growth retardation and aberrant body composition [12]. Despite recommendations for term infants being unable to be applied to preterm infants due to their peculiar nutritional needs and neurodevelopmental trajectories, including feeding difficulties, no universal guidelines for preterm infants’ complementary foods introduction are available [7,12,13]. Given the critical role of micronutrients in supporting adequate growth and neurodevelopment [14,15], as listed in Table 1, attention to their intake should also be paid during complementary food introduction, especially when acceptance of semi-solid food is still incomplete or problematic [7]. Previous feeding history (exclusively breastfeeding, fortification, use of post-discharge or standard formulas), growth patterns, including the achievement of catch-up growth, and the presence of eating difficulties should also be taken into consideration when planning complementary foods introduction in preterm infants [7]. Preterm infants are supplemented with iron and multivitamins after discharge, but the length and dose of supplementation widely differ [16].

This narrative review aims to update current knowledge on the iron, vitamin D, zinc, calcium, phosphorus and long-chain polyunsaturated fatty acids (LCPUFA) supplementation of preterm infants during complementary feeding. We choose to focus on these specific micronutrients in light of their importance in contributing to preterm infants’ outcomes.

## 2. Iron

Iron is considered an essential nutrient as it plays a crucial role in hemoglobin (Hb) synthesis and oxygen transport and it is involved in several enzymatic processes, particularly in cellular energy production [17]. The majority of the iron supply accumulates during the last trimester of pregnancy due to active transport in the placenta. The total iron content in the term newborn is approximately 75 mg/kg: the most significant amount (75–80%) is included in the Hb, approximately 10% in tissues is in iron-containing proteins (myoglobin and cytochromes), and the remaining 10–15% represents storage iron (ferritin and hemosiderin) [17]. Iron storage at birth depends on both prenatal factors, such as the maternal iron status and the possibility of iron transfer to the fetus and perinatal factors, such as cord clamping. Delayed cord clamping has been suggested to increase the perinatal transfer of Hb and improve the term and preterm infants’ iron status [18]. Proper iron status at birth is essential to support iron homeostasis in the first 6–9 months of life when a physiological decrease occurs, due to insufficient absorption and the relatively low content in breast milk [19].

Since prematurity ceases the placental transfer, the preterm infants’ iron stores at birth are decreased proportionally to the gestational age and the birth weight [20]. A further reduction in the fetal iron storage may be related to maternal iron deficiency anemia, maternal hypertension, diabetes and intrauterine growth retardation [20,21]. Other pregnancy-related factors such as multiple pregnancies and obesity may further decrease the iron stores of the preterm infant [22]. In fact, all these conditions, often associated with premature birth, can lead to a condition of chronic placental insufficiency and therefore decreased prenatal iron transfer. Furthermore, the rapid recovery of growth typical of premature babies, the need to initiate erythropoiesis earlier in development and frequent infusions can further reduce the body’s iron stores [23].

At hospital discharge, the iron deposit shows a significant variability according to the course of the Neonatal Intensive Care Unit (NICU) stay, including the timing and dose of enteral iron supplementation, dietary intake, the development of extrauterine growth retardation, the erythropoietin administration and concentrated red blood cell transfusions [17,20,24]. Post-discharge supplementation, maintained after the introduction of complementary feeding, requires careful adjustment based on the positive or negative iron balance [17,20,24]. The recommended dietary iron intake is 2–3 mg/kg/day for infants with a birth weight of <1500 g, 2 mg/kg/day for infants with a birth weight of 1500–2500 g and 1–2 mg/kg/day for infants with a birth weight > 2500 g [20]. However, it is worthwhile mentioning that the iron requirement should be tailored accordingly not only to the birth weight and the degree of prematurity but also to the type of feeding, the need for catch up growth due to the prompt adjustment of the circulating red blood cells and to the level of Hb and ferritin [20,25]. In very low birth weight (VLBW) infants with ferritin levels < 35 ug/L, particularly if breastfed, the dietary iron intake must be increased up to 3–4 (max. 6) mg/kg/day; however, in the case of ferritin levels > 300 ug/L, which can be a common finding after blood transfusions, iron supplementation should be postponed [25]. In fact, it must be taken into account that premature infants are vulnerable to oxidative stress caused by iron overload [26]. This susceptibility is due to the immature antioxidant system. The overload could be caused by high doses of oral or parenteral iron supplementation and recurrent erythrocyte transfusions [22]. Blood transfusions could be a relevant risk for premature infants, considering that approximately 40% of VLBW and 90% of extremely low birth weight (ELBW) infants receive at least one blood transfusion during their hospital stay [27]. In the last years, there has been a shared call for more restrictive blood transfusion policies in particular for the extremely preterm neonates, because of the demonstrated associations between early exposure to blood transfusions, and increased mortality and short-term morbidities [28].

Within this context, when planning iron supplementation at discharge, the clinical history of the preterm infant should be taken into account, from the number and date of blood transfusions received to the type of feeding. Exclusive human milk feeding does not meet such high iron intakes if not either fortified or complemented with post-discharge formula milk, currently providing 2.25 mg/kg of iron if consumed at 150 mL/kg/day [20]. When introducing complementary foods in preterm infants, offering animal source foods containing heme iron could represent a strategy to increase iron intake, especially in breastfed infants born extremely preterm who have not achieved catch-up growth yet. Families willing to apply a vegetarian or a vegan diet during the complementary feeding period should be offered a specialistic input to balance this kind of diet at most [29].

From a practical perspective, given the increased risk of iron deficiency associated with prematurity and low birth weight, we suggest monitoring the iron storage status at discharge, during follow-up and at the beginning of complementary feeding in order to tailor iron supplementation dose and duration and offer the most appropriate solid foods. Periodic monitoring of iron homeostasis is necessary since iron overload from excessive or unnecessary supplementation may lead to oxidative stress with a subsequent increased risk of infections and growth retardation and retinopathy of prematurity development [17,30]. A complete hemocromocytometric analysis enables an evaluation of the degree of anemia of prematurity and the differentiation amongst microcytic, normocytic and macrocytic anemia based on the mean corpuscular volume [31]. However, hemoglobin reduction represents a late sign of iron deficiency and becomes apparent once the adverse effect on cognitive development has already occurred [32,33]. The most sensible method to examine the neonatal iron status is represented by the ferritin level, which will serve when evaluating the iron supplementation’s efficacy in the first year of life [17,18,20,21] (Table 2).

## 3. Zinc

Zinc plays a crucial role in the various cellular phases, through a catalytic, structural and regulatory function. It is essential for tissue integrity, especially at the gastro-intestinal and respiratory level and contributes to immunomodulation (phagocytosis and cellular immunity) [34], bone development, growth hormone regulation, gustative function and appetite [35,36,37]. The regular intake of zinc is critical since the body cannot produce zinc and does not have an appropriate system for storing and releasing it [36,38]. Zinc content gradually decreases through the first months of lactation ranging from 8–12 mg/L in colostrum to 1–3 mg/L at one month of life [39]. Remarkably, zinc concentration in preterm human milk has been reported to be significantly lower than that in term milk through the first two months of corrected age [17,40]. Formula milk concentration of zinc ranges from 1.5 to 6 mg/L; however, zinc net absorption with formula is significantly lower than with human milk (60 vs 20%) [39].

In preterm infants, the blood zinc concentration decreases during the first months of life, leading to a zinc level lower in the preterm newborn at 40 weeks post-conceptional age than the term neonate [39]. The diagnosis of zinc deficiency is not straightforward due to the lack of accurate biomarkers of zinc status. Although it presents some limitations, serum zinc concentration assessment remains the best marker to identify zinc deficiency [39,41]. There is no homogeneous advice concerning dose, frequency and length of the prophylactic zinc supplementation [42]. It must be kept in mind that, when supplemented, zinc can interfere with other microelements, for example, influencing the iron and copper absorption and vice versa [43]. The process through which zinc may lead to anemia and copper deficiency is not entirely understood [44]. A study by Griffin et al. described how at the zinc intakes suggested by the authors (1.8–2.4 mg/kg/d in formula-fed infants and 2.3–2.4 mg/kg/d in breastfed infants), copper intakes need to be increased up to 250 mcg/kg/d to maintain adequate copper retention [45].

Since zinc does not have pro-oxidant properties and rarely can cause side effects from an overdose [20,32], based on the available evidence [45], it can be suggested to provide zinc supplementation through the first year of life. In particular, zinc supplementation should be considered in breastfed preterm infants experiencing impaired growth [46]. We do not recommend serial measurements of its concentration unless evidence of zinc deficiency is detectable (Table 2).

## 4. Calcium and Phosphorus

Most of the bone mineralization occurs in the third trimester of pregnancy [47]. In this period, in fact, 80% of the bone mineral stock is deposited by the action of osteoblasts. This process is strictly dependent on the availability of calcium and phosphorus via active placental transport, that leads to a physiological “fetal hypercalcemia”, essential for bone formation. The placenta transfers to the fetus 120 mg/kg/day of calcium and 60 mg/kg/day of phosphorus [48]. At birth, the onset of respiratory activity increases blood pH, causing a reduction in circulating calcium ions and a rise in parathormone levels. This process is accompanied by the initiation of a mineral resorption from the bone, causing a decrease in bone mineral density that persists up to 6 months of age [49]. In term infants, however, bone integrity is maintained. On the contrary, premature babies are at great risk of osteopenia. Premature birth places an abrupt halt to this process, effectively blocking bone mineral accumulation. At birth, the transition to extrauterine life worsens this deficiency. In fact, in addition to the mechanisms already described for the term newborn, in the preterm the absorption of calcium is further compromised by poor gastrointestinal tolerance and motility, as well as by the use of drugs that increase its excretion, such as diuretics (furosemide) and theophylline [49]. The sum of these risk factors explains the increased susceptibility of preterms to metabolic bone disease (MBD), a disorder characterized by hypophosphatemia, hyperphosphatasemia and, at later stages, radiological findings of bone demineralization or clinical features such as softening or fractures of ribs and other bones, enlargement of the cranial sutures, frontal bossing and rickets [50]. It has been reported that MBD occurs in 55% of neonates with extremely low birth weight (ELBW ≤ 1000 g birth weight) and 23% of infants with very low birth weight (VLBW < 1500 g). In parallel, it appears to be more frequent in extremely premature babies, born before 28 weeks of gestation [51]. The mode of feeding and supplementation can also influence the development of MBD. In breastfed premature infants the incidence is 40%, and in formula-fed infants that undergo oral calcium and phosphorus supplementation, the incidence is reduced to 16%. However, it must be kept in mind that the absorption of calcium in formula-fed infants is 40%, and 70% in breastfed infants [51]. Since clinical findings of MBD appear in later stages of the disease, the assessment of serum biochemical markers is a useful screening tool for preterms at risk for mineral deficiency. Although there are no biochemical markers specific for MBD of prematurity, the bone metabolism can be assessed by dosing calcium, phosphate, alkaline phosphatase (ALP), parathormone (PTH) and vitamin D in the first weeks of life, especially in high-risk patients such as VLBW infants. Urinary calcium and phosphate excretion, together with their creatinine ratios, have also been indicated as biomarkers of postnatal skeletal mineralization. The MBD should be most of all prevented, by ensuring adequate calcium, phosphate and vitamin D intake and limiting the use of specific medications, such as diuretics and methylxanthines that reduce mineral stores and glucocorticoids which enhance bone resorption [52]. In infants receiving total parenteral nutrition (TPN), calcium and phosphorus intakes are usually gradually increased from 40–120 to 75–90 mg/kg/day for calcium and 31–71 to 60–70 mg/kg/day as the ml/kg ratio of TPN increases. Regarding infants in full enteral feeding, recommendations suggest an oral supplementation of 140–160 mg/100 kcal (for the American Academy of Pediatrics, AAP) [53] or 70–140 mg/100 kcal (for the European Society for Pediatric Gastroenterology, Hepatology and Nutrition, ESPGHAN) of calcium and a supplementation of 95–108 mg/100 kcal (AAP) or 50–86 mg/100 kcal (ESPGHAN) of phosphorus [54]. Preterm formulas or breast milk fortification are indicated until discharge, whilst there is no consensus on their use after the hospital stay. In VLBW infants, an assessment of bone metabolism biomarkers by 2–4 weeks post-discharge has been suggested. If ALP values are equal to or above 800–1000 UI/mL, calcium and phosphate supplementation should be initiated or increased [50]. There is still no consensus on the duration of this mineral supplementation. However, it must be kept in mind that growth-faltering infants may need to be supplemented up to 6 months of age, and breastfed preterm infants can reach the adequate bone mineralization only at two years of life. Considering the importance of these two minerals for the health of the premature baby, and the increased risk of developing MBD, it seems reasonable to suggest the assessment of the metabolic status of the bone through blood tests performed during the follow-up after discharge. This can lead to an early diagnosis of osteopenia and therefore to the initiation of adequate supplementation, and it can also help in identifying those children who, due to dietary variations or concomitant pathologies and drugs, may require greater or more prolonged supplementation.

## 5. Vitamin D

In the last years, the role of vitamin D and its supplementation have become more and more critical. Not only has its well-known activity on the bone mineralization been thoroughly examined, but also new metabolic functions, defined as “extra-skeletal”, have been evaluated [55]. The research has then focused on the extra-skeletal effects of this vitamin. In some studies vitamin D has been categorized as a hormone. Researchers have described its ability to regulate over 1250 genes’ expression [56]. In fact, among the various functions recently discovered we find anti-tumor actions [57], regulation of the inflammatory function [58] and protection against cardiovascular risk factors [59] through innumerous metabolic pathways, still not fully understood [57]. The majority (>90%) of vitamin D is synthesized by skin exposition to sunlight, whereas the intake from diet is less significative. Although vitamin D synthesis from sunlight exposure is highly effective, the sunlight hours are reduced during the cold season, thus impairing the vitamin D production [60]. Moreover, the American Academy of Pediatrics (AAP) does not recommend to expose children less than six months of age directly to the sunlight without proper clothing, reducing sunlight exposure and vitamin D synthesis [61]. Epidemiological studies have underlined a high prevalence of hypovitaminosis D (>50%) during infancy [55]. This inadequacy of vitamin D can be defined as insufficiency when the level of 25-OH-D is between 20 and 30 ng/mL, and as a deficiency when the serum concentration is < 20 ng/mL [62].

The first year of life represents a crucial time to prevent the vitamin D deficit. The neonatal storage of vitamin D is directly related to the maternal vitamin D status, as shown by the positive link between the maternal and the cordonal or neonatal levels of 25-OH-D [63], and may depend on the maternal supplementation, the ethnic origins and the birth season [62]. The importance of maternal vitamin D status has been further highlighted by a recent study by Treiber et al.: the authors have demonstrated that a severe deficiency of vitamin D in cord blood is significantly associated with an increased risk of preterm birth, neonatal respiratory distress syndrome and an increased risk of hospitalization during the first year of life for acute infections such as respiratory infections or gastroenteritis [64]. In light of newborns’ unique rapid growth, they can be more subject to a vitamin D deficiency, especially if they are exclusively breastmilk-fed, since human milk has inadequate vitamin D levels (<80 UI/L) to prevent deficiency status. Moreover, formula milk-feeding alone even when fortified with 400 UI/L of vitamin D, may lead to an inadequate level of vitamin D. In fact, based on the vitamin D content in formula milk, it is estimated that it is necessary to take about one litre of milk per day, which in general occurs when the infant reaches approximately a weight of 5–6 Kg, which means after 2–3 months for term babies, but even later in preterm or small for gestational age infants [55]. Therefore, in the term born without risk factors, although rickets is easily prevented, it is still unclear whether supplementation can cover any subclinical deficiencies at a systemic level, considering the endocrine actions of vitamin D. While it is difficult for the full-term baby to have a sufficient vitamin D supply, it is even more so for the preterm baby. First of all, a possible lengthy hospitalization poses an obstacle to sun exposure. Secondly, due to the metabolic characteristics of preterm babies, vitamin D requirements may be higher. The majority of the bone mineralization physiologically happens during the last trimester of pregnancy, especially between the 32nd and 36th post-menstrual weeks [65]. The premature baby does not access the third trimester large supply of these two minerals (approximately 80% of the total for the entire duration of gestation), thus being born in a condition of reduced mineral reserves. Moreover, given the particular immaturity of the digestive and renal systems, the absorption of calcium and phosphorus from nutritional sources is also reduced, while their renal loss is increased. Therefore, a vitamin D deficiency in premature babies further worsens these minerals’ absorption and availability [66]. Additionally, various pharmacological therapies, currently in neonatal intensive care, like diuretics or steroids, and the total parenteral nutrition can impact already delicate bone mineralization [65]. A recent study by Zhang et al. demonstrated the importance of vitamin D status in preterm infants. In this observational study, the authors described a significant correlation between the mean serum 25(OH)D level (less than 25 nmol/L, 25–50 nmol/L, and more than or equal to 50 nmol/L) and the occurrence of neonatal respiratory distress syndrome (RDS) rates among the three groups [67].

Overall, in the literature, a specific time for checking the 25-OH-D levels has not been established yet, both for the term and preterm infants. Currently, the routinary dosage after and during the prophylaxis is not recommended, and this should be reserved, with annual frequency, to those babies with specific risk factors such as non-Caucasian ethnicity with hyper-pigmented skin, chronic liver insufficiency, chronic kidney disease, malabsorption or being on medications which can interfere with the vitamin D metabolism [65,66]. The neonatal vitamin D level is around 50–70% of the maternal blood level, both in term and preterm births. Research in recent years has therefore focused a lot on preventing vitamin D deficiency in pregnant women. However, to date, there are no infant supplementation protocols based on the often unknown maternal status. Moreover, studies on vitamin D status in newborns and functional biomarkers such as intestinal calcium absorption, bone mineralization and PTH concentrations are lacking. Therefore, vitamin D prophylaxis is the safest and most effective way to prevent a vitamin D deficit and must be implemented for the whole first year of life in all infants, regardless of the type of milk feeding. This simple measure will prevent children from developing rickets, which is the extreme consequence of hypovitaminosis D. A recent meta-analysis including 61 studies and 1828 patients demonstrated the effectiveness of the 400 UI/day supplementation in preventing nutritional rickets and assuring adequate 25-OH-D serum concentrations [68].

Since the neonatal storage relates to the maternal status and the half-life of vitamin D is around 2–3 weeks, the prophylaxis must be commenced from the first few days of life. When preterm infants reach the term corrected age (40 weeks of gestational age), most of the Neonatal and Pediatric Societies advise to continue the same recommended dose of a term neonate (400 UI/day), as shown in Table 3.

In the presence of the already described risk factors, the supplementation with higher dosage may be recommended (up to 1000 UI/day of vitamin D), especially during months with reduced sunlight [62]. The administration of vitamin D2 or D3 is equally effective, whereas the metabolites or analogues like calcifediol, alfa-calcidiol, calcitriol or dihydrotachysterol must not be used as a prophylactic measure, except for some specific conditions. These metabolites may lead to hypercalcemia, and they are unable to keep and restore the vitamin D reserve [62]. A precise evaluation of hypovitaminosis D’s risk factors can be a valuable screening measure to identify a possible deficiency status and promptly begin the recommended prophylaxis. Considering that even higher doses of vitamin D do not show a significant risk of side effects and that an overdose is an infrequent event, vitamin D supplementation should be started in the first days of life regardless of the type of feeding and continued well beyond the neonatal age [55] (Table 2).

## 6. LCPUFA

Docosahexaenoic acid (DHA) is the most represented n-3 LCPUFA, whereas arachidonic acid (AA) constitutes most common n-6 LCPUFA. During fetal life, the placenta promotes the DHA and AA transport, thus favoring their accumulation, which reaches its maximum during the last trimester of pregnancy. Overall, the accretion of DHA during fetal life occurs mainly in the third trimester, and is estimated to be 10 g [72]. At birth, the levels of DHA and AA are significantly correlated with the gestational age; therefore, babies born the earliest are at the highest risk for deficiency [73]. Consequently, prematurity poses a significant risk of LCPUFA deficit [74]. Following delivery, infants rely on enteral sources to meet their DHA requirement, whether through breast milk or formula. LCPUFA levels in breastmilk are related to the daily, or weekly dietary intake of DHA, which appears to be a determining factor for its milk content, rather than maternal deposits [75]. Regardless of maternal deposits, the highest DHA levels were found in those populations that regularly consumed fishery products [76]. The content of LCPUFA and DHA in breast milk, in particular, is influenced not only by the maternal dietary intake but also by other factors, such as cigarette smoking, with a dose-dependent mechanism. Some studies have shown that the progressive decrease in fetal DHA values, which is associated with maternal smoking in pregnancy, is accompanied by a parallel increase in the metabolic precursor of DHA, alpha-linolenic acid (ALA), explaining how the direct intake of docosahexaenoic acid is essential, especially by smoking mothers [77].

In the last 20 years, several studies have focused on the effects of LCPUFA supplementation on vision and neurodevelopment. On one side, some meta-analyses showed no significant impact on the long-term visual and cognitive outcomes [74,78,79]. In the most recent review by Gould et al. that involved 25 randomized controlled trials correlating DHA interventions with assessments of behavioral functioning in childhood, none of the studies demonstrated a beneficial effect of the intervention on the infants’ neurodevelopment [80]. Moreover, two trials detected adverse effects. On the other side, various studies highlight that LCPUFA may be beneficial for the preterm newborns. A meta-analysis reviewed various controlled trials of n-3 polyunsaturated fatty acid (PUFA) supplementation in infants (≤2 years of age) and mothers and assessed the visual and cognitive development up to 18 years of age through standardized scales. It was underlined that n-3 supplementation positively affected the visual and psychomotor functions (an increase in the Mental Developmental Index of three scores according to the Bayley Scale) of the preterm neonates [81]. In addition, a recent study by Hellström et al. demonstrated that AA and DHA supplementation reduced severe ROP (Retinopathy Of Prematurity) incidence by 50% [82]. Given the conflicting results of the listed studies, it is currently difficult to give clear indications on the benefits of LCPUFA supplementation in preterm infants. To date, there is no international consensus on the benefits of adding DHA and AA to infant formulas, both for term and preterm infants. The European Commission is planning to indicate DHA as a mandatory nutrient in infants formulas, at the latest in February 2021 [83]. However, the DHA and AA ration appears be more significant than supplementing DHA alone. Formula-fed babies, therefore, should not be considered protected from LCPUFAs deficiency.

Premature infants are typically deficient in DHA for several reasons, including their inability to convert large amounts of precursor fatty acids to DHA, due to their metabolic immaturity, combined with their high requirements [84]. De Rooy et al. published a study that evaluated DHA levels in extremely premature infants receiving standard care. This longitudinal study evaluated omega-6 and omega-3 intake from all sources in infants born before 28 weeks of Gestational Age. The authors showed that the total amount of DHA that infants received at the end of 6 weeks represented only 36.6% of DHA that they would have received in utero [85]. Premature infants are at risk for inflammatory diseases such as bronchopulmonary dysplasia (BPD) and necrotizing enterocolitis (NEC). Docosahexaenoic acid is already known for its anti-inflammatory properties [84]. Based on these previous findings the N-3 Fatty Acids for Improvement in Respiratory Outcomes (N3RO) study was developed. This large, blinded, controlled, randomized trial assessed the effect of DHA given to premature infants born before 29 weeks on BPD incidence. Surprisingly, the authors concluded that supplementing premature infants with DHA could actually have increased the risk of developing BPD [85]. Prematurely born children are at higher risk of autistic spectrum disorder (ASD). Considering that fatty acids are related to cognitive functions, some trials have recently focused on the role of LCPUFA in the ASD. They demonstrated that the combination of n-3 and n-6 fatty acids (including the γ-linolenic acid, GLA) might lead to less severe ASD symptoms [86].

The postnatal supplementation of LCPUFA on the visual acuity and cognitive development in the moderately preterm neonates (MPT, 30–36 weeks of gestational age) appears to have possible advantages in neurodevelopment year of age when supplementing the formula milk with both DHA and AA [87]. Regardless of their gestational age, low birth weight infants are already more susceptible to developing behavioral and mental issues. Although DHA and AA requirements in late and moderately preterm newborns are probably higher than in term infants, there is no sufficient evidence to advise specific doses [88]. Based on the well-documented benefits of LCPUFAs in preterm infants, and considering the described deficiency in premature babies, we believe it could be useful to supplement DHA in premature babies. This could be achieved both through the use of dedicated supplements or, in the absence of breast milk, through DHA-enriched infant formulas (Table 2).

## 7. Conclusions

Prematurity implies a high risk of morbidity and mortality. Early adequate nutritional support of preterms is paramount to limit adverse metabolic outcomes.

To date, there is no internationally shared agreement on supplementation of iron, zinc, calcium, phosphate, vitamin D and LCPUFAs in the premature baby after discharge and in the first year of life. However, the current literature shows little to no adverse effects deriving from their supplementation. In addition, the knowledge of these micronutrients is increasing, and more and more positive systemic effects of these substances are described.

In our opinion it seems fair to say that providing these supplements to premature babies can bring more benefits than risks. This is especially true for the breastfed premature infant. Although breast milk represents the best alternative for this population, it is clear how the content of some micronutrients, in particular LCPUFAs, is strictly dependent on maternal deposits and nutrition. This review highlights the knowledge gap existing in the literature on premature supplementation, and the need for large randomized trials to address these issues.

## Figures and Tables

**Table 1:** Functional effects of iron, zinc, vitamin D, calcium, phosphorus and long-chain polyunsaturated fatty acids (LCPUFAs) and populations subgroups at risk for inadequate intake.

	Functions	High Risk Population Subgroups
**Iron**	Hemoglobin synthesis; Oxygen transport; Cellular energy production	Very low birth weight infants Preterms < 34 GA Intrauterine growth restriction Preterms fed non-fortified breastmilk during hospital stay
**Zinc**	Tissue integrity; Immunomodulation; Bone development; Growth hormone regulation; Appetite regulation	
**Vitamin D**	Bone mineralization; Anti-tumor actions; Inflammatory functions Cardiovascular functions	
**LCPUFAs**	Retinal development; Neurodevelopment	
**Calcium and Phosphorus**	Bone mineralization; Neuromuscular function	

GA: Gestational Age.

**Table 2:** Summary of recommendations regarding the measurement and supplementation of iron, zinc, vitamin D, calcium, phosphorus and LCPUFAs in discharged infants in the first year of life.

	Measurements	Supplementation
**Iron**	Measurement of iron storage status is recommended: -at discharge -during follow-up -at the beginning of complementary feeding	Iron supplementation should be tailored according to: -birth weight -gestational age -type of feeding -need for catch up growth -iron status
**Zinc**	Serial measurements of zinc concentration are not recommended unless evidence of zinc deficiency is detectable	Zinc supplementation through the first year of life may be advisable, particularly in breastfed infants with impaired growth
**Vitamin D**	Serial measurements of Vitamin D are not recommended, unless specific risk factors are identified	Vitamin D supplementation is recommended at least up to the first year of life
**LCPUFAs**	Serial measurements of LCPUFA, particularly DHA and AA, are not recommended	DHA supplementation may be advisable but there is no sufficient evidence to advise specific timing and doses
**Calcium and Phosphorus**	Serial measurements of serum calcium, phosphate, alkaline phosphatase (ALP), parathormone (PTH) and vitamin D are not recommended but may be advisable in VLBW infants in the first weeks after discharge. Assessment of urinary calcium and phosphate to creatinine ratios may be useful.	Calcium 140–160 mg/100 kcal (AAP) 70–140 mg/100 kcal (ESPGHAN) Phosphorus 95–108 mg/100 kcal (AAP) 50–86 mg/100 kcal (ESPGHAN)

AAP: American Academy of Pediatrics; ESPGHAN: European Society for Pediatric Gastroenterology, Hepatology and Nutrition; VLBW: Very Low Birth Weight.

**Table 3:** Summary of Vitamin D supplementation in preterm infants up to term corrected age [17,23,24,25,26].

AAP 2013 [60]	ESPGHAN 2010 [69]	Other Guidelines [70,71]
Birth weight < 1500 g: 200–400 IU/day Birth weight > 1500 g: 400–1000 IU/day	800–1000 UI/day	400–800 UI/day

AAP: American Academy of Pediatrics; g: grams; UI: International Units.
